# Immunohistochemical Expression Pattern of FGFR1, FGFR2, RIP5, and HIP2 in Developing and Postnatal Kidneys of *Dab1^−/−^* (*yotari*) Mice

**DOI:** 10.3390/ijms23042025

**Published:** 2022-02-11

**Authors:** Nela Kelam, Anita Racetin, Yu Katsuyama, Katarina Vukojević, Sandra Kostić

**Affiliations:** 1 Department of Anatomy, Histology and Embryology, University of Split School of Medicine, Šoltanska 2, 21000 Split, Croatia; nela.kelam@mefst.hr (N.K.); amuic@mefst.hr (A.R.); sandra.kostic@mefst.hr (S.K.); 2 Department of Medical Genetics, University of Mostar School of Medicine, 88000 Mostar, Bosnia and Herzegovina; 3 Department of Anatomy, Shiga University of Medical Science, Ötsu 520-2192, Japan; kats@belle.shiga-med.ac.jp

**Keywords:** RIP5, FGFR1, FGFR2, HIP2, Erk1/2, mTOR, kidney development, *yotari* mice

## Abstract

This study aimed to explore how *Dab1* gene functional silencing influences the spatial and temporal expression patterns of fibroblast growth factor receptor 1 (FGFR1), fibroblast growth factor receptor 2 (FGFR2), receptor-interacting protein kinase 5 (RIP5), and huntingtin-interacting protein 2 (HIP2) in the developing and postnatal kidneys of the *yotari* mice as potential determinants of normal kidney formation and function. *Dab1^−/−^* animal kidneys exhibit diminished FGFR1/FGFR2 expression in all examined developmental stages, whereas RIP5 cell immunoreactivity demonstrated negligible variation. The HIP2 expression revealed a discernible difference during the postnatal period, where we noted a significant decrease in almost all the observed kidney structures of *yotari* animals. An extracellular signal-regulated kinase (Erk1/2) and mammalian target of rapamycin (mTOR) expression in *yotari* kidneys decreased in embryonic and postnatal developmental phases for which we can hypothesize that the Erk1/2 signaling pathway in the *yotari* mice kidneys is dependent on Reelin with Dab1 only partially implicated in Reelin-mediated MEK/Erk1/2 activation. The impairment of FGFR1 and FGFR2 expression suggests the involvement of the observed markers in generating the CAKUT phenotype resulting in renal hypoplasia. Our study demonstrates the critical role of HIP2 in reducing cell death throughout nephrogenesis and maturation in wild-type mice and indicates a possible connection between decreased HIP2 expression in postnatal kidney structures and observed podocyte injury in *yotari*. Our results emphasize the crucial function of the examined markers throughout normal kidney development and their potential participation in kidney pathology and diagnostics, where they might serve as biomarkers and therapeutic targets.

## 1. Introduction

The *yotari* (*Dab1^−/−^*) mouse, an autosomal recessive mutant mouse, arose spontaneously during the generation of mice carrying a gene mutation encoding the receptor for inositol-1,4,5-trisphosphate [1]. The phenotype of the *yotari*, very similar to those of *reeler* (*Reelin^−/−^*) mice, is characterized by unsteady gait, tremors, and premature death at the time of weaning [1]. Our latest research revealed the congenital anomalies of the kidney and urinary tract (CAKUT) phenotype resulting in renal hypoplasia followed by foot process effacement in the kidney glomeruli and loss of functional kidney tissue of *yotari* [2]. The data implicate chronic kidney disease (CKD) as the cause of *yotari* mice’s premature death, which can be propagated by various mechanisms that may influence the kidney structures [2,3]. The Disabled-1 (Dab1) protein has been found in mouse podocytes [4], human fetal kidneys [5] and functions as an adapter molecule of intracellular signal transmission [6,7].

Morphological renal aberrations in humans that are part of the CAKUT spectrum [8,9] are related to mutations in fibroblast growth factor receptors (FGFRs) and substrates on their signaling pathways [10], one of them being receptor-interacting protein kinase 5, RIP5 (DSTYK) [11].

Expressed in the cell membranes of maturing epithelia of the kidney, RIP5 colocalizes with FGFRs in the ureteric bud and metanephric mesenchyme, thus making it a significant determinant of human urinary tract development downstream of fibroblast growth factor (FGF) signaling [11,12,13]. RIP5 knockdown in human embryonic renal cells blocked FGF-stimulated phosphorylation of extracellular signal-regulated kinase (Erk), the paramount signal downstream of receptor tyrosine kinases [11]. Additionally, independent RIP5 mutations were detected in 2.3% of patients with CAKUT, supporting its role as a critical regulator of proper renal development [11].

The FGF/FGFR signaling cascade regulates several essential biological processes such as embryonic development, angiogenesis, and tissue regeneration, which is required to pattern virtually all renal lineages during the early and late stages of development [14,15]. Mitogen-activated protein kinases (Ras/Raf-MEK-MAPKs) and phosphatidylinositol-3 kinase/protein kinase B/mammalian target of rapamycin (PI3K/AKT/mTOR) are some of the most well-known downstream signaling pathways for FGF/FGFR [16,17,18]. A number of studies support an important role for Erk1/2 as a mediator of Fgf signaling in many biological processes [19].

The impairment of the FGF/FGFR signaling axis is observed in various human syndromes and diseases, such as CKD, diabetes, and multiple tumors [11,14,20]. In diabetes, the rising evidence highlights the protective implications of FGFR1 and endothelial FGFR1 [21]. It has been shown that FGFR1 increases the levels of renal protective microRNAs, which play a critical role in kidney development [22]. It is well known that some bioavailable peptides protect the kidneys by increasing FGFR1 expression or suppressing inflammatory cytokines [23,24,25].

Many genetic *Fgfr* and *Fgf* animal models have structural defects mimicking numerous CAKUT seen in humans [20,26,27,28,29,30]. In mice, deletion of *Fgfr1* or Fibroblast growth factor receptor 2 (*Fgfr2*) results in embryonic lethality before the onset of renal development [15,31,32,33,34]. Global deletion of *Fgfr1* interrupts nephron formation, while loss of *Fgfr2* in the metanephric mesenchyme, critical for ureteric morphogenesis, leads to many kidney and urinary tract anomalies [28]. After birth, renal hypoplasia in Fgfr2 mutants resulted in CKD, hypertension, and left ventricular hypertrophy [35]. Transgenic mice with a dominant-negative *Fgfr* fragment establish renal aplasia or severe dysplasia [36].

Our recent studies displayed that huntingtin-interacting protein 2 (HIP2), co-expressed with RIP5, is strongly expressed in glomeruli, the collecting system, and the urothelium of the developing human kidney [13].

This report aimed to analyze how *Dab1* gene functional silencing influences the expression and localization of RIP5, FGFR1, FGFR2, HIP2, mTOR, and Erk1/2 in developing and postnatal kidneys of *Dab1^−/−^* (*yotari*) mice. Our study analyzes similar phenotypic features, the effects of *Dab1* and *Fgfr1* inactivation in kidney tissues to investigate whether *Dab1* inactivation disrupts the FGFR1 signaling system. We hypothesize that these proteins are expressed in the developing and postnatal mouse kidneys, and their functional interplay contributes to preserving their structure and function. Results of our study might have meaningful implications in better understanding the normal mammalian kidney development and possibly in suggesting the therapeutic potential of investigated markers.

## 2. Results

The immunoexpression of FGFR1, FGFR2, and HIP2 was analyzed on metanephric mesenchyme (mm), renal vesicles (rv), glomeruli (g), convoluted tubules (Ct), ampullae (A), and collecting ducts (Cd) at embryonic days E13.5 and E15.5, and glomeruli (G), proximal convoluted tubules (PCT), and distal convoluted tubules (DCT) at postnatal days P4, P11, and P14 in *yotari* (*Dab1^−/−^*) and wild-type (wt) mouse kidneys, with a particular focus on co-expression with RIP5, expressed in the cell membranes of maturing epithelia. Erk1/2 and mTOR were also examined to make an assumption about downstream signaling pathways. Vimentin was used as a marker for cells of mesenchymal origin and nephrin as a marker of podocyte differentiation and function as presented in Supplementary Figure S1.

### 2.1. FGFR1 Expression

**Figure 1:**
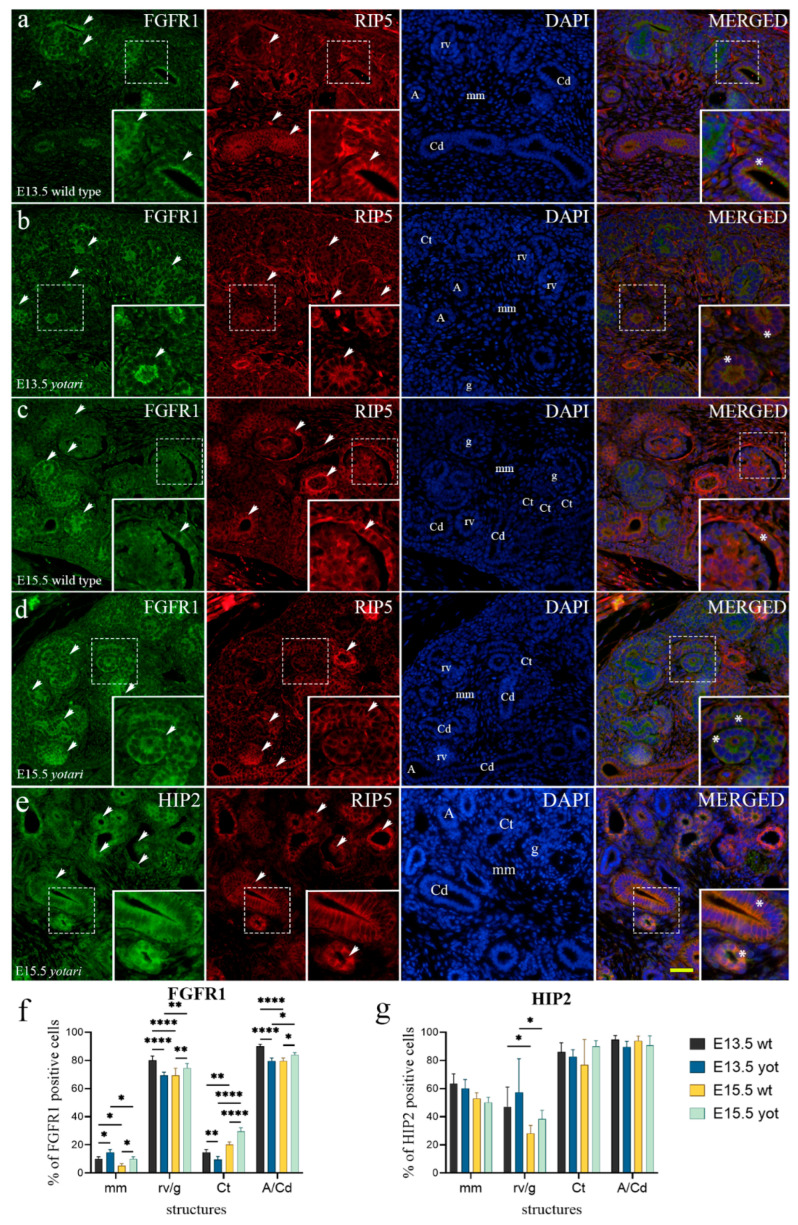
Double immunofluorescence staining of embryonic wild-type and *yotari* mouse kidneys with the FGFR1 (**a**–**d**), HIP2 (**e**), and RIP5 markers (**a**–**e**). Arrows show the expression pattern of FGFR1, HIP2, and RIP5 in metanephric mesenchyme (mm), renal vesicles (rv), glomeruli (g), convoluted tubules (Ct), ampullae (A), and collecting ducts (Cd) indicated on 4′,6-diamidino-2-phenylindole (DAPI) image. Immunoexpression of FGFR1, RIP5, DAPI staining and merged FGFR1, RIP5, and DAPI at embryonic days E13.5 and E15.5 in wild type (**a**,**c**) and *yotari* (**b**,**d**). Immunoexpression of HIP2, RIP5, DAPI staining and merged HIP2, RIP5, and DAPI at E15.5 in *yotari* (**e**). Wild-type and *yotari* mice at E13.5 of kidney development mostly correspond regarding localization and intensity of HIP2; thus, a representative image was selected from E15.5. The most prominent protein expression area is shown in inserts, corresponding to the dashed boxes. An asterisk denotes the zone where co-expression was detected. Images were taken on magnification ×40. The scale bar is 50 μm, which refers to all images. The distribution of the percentages of FGFR1 (**f**), and HIP2 (**g**) positive cells in the metanephric mesenchyme (mm), renal vesicles (rv) or glomeruli (g), convoluted tubules (Ct), and ampulla (A) or collecting ducts (Cd) of wild type and *yotari* kidneys at embryonic days E13.5 and E15.5. Data are presented as the mean ± SD (vertical line) and analyzed by a two-way ANOVA test followed by Tukey’s multiple comparison test. Significant differences were indicated by * *p* < 0.05, ** *p* < 0.001, **** *p* < 0.00001. At each time point, ten substructures were assessed.

At E13.5, strong punctate expression of FGFR1 was observed within the apical membrane of collecting ducts, including ampullae and developing nephrons (metanephric cup, renal vesicle stages), but weakly in the undifferentiated cells of metanephric mesenchyme (interstitium) and convoluted tubules of wt mice (Figure 1a and Figure S2a,b). In the surrounding metanephric mesenchyme, cells close to the collecting duct predominantly expressed FGFR1, while towards the periphery, the expression had a reverse pattern (Figure 1a). The same pattern was observed in the *yotari* mice, with significant, slightly elevated expression levels in metanephric mesenchyme, decreasing significantly in all other substructures (*p* < 0.05, Figure 1b,f and Figure S3a).

Semi-quantitative analysis of both animal genotypes revealed mild staining intensity in metanephric mesenchyme and convoluted tubules and moderate reactivity in mitotic cells of renal vesicle stages and collecting ducts (Table 1).

The co-expression of the two markers, FGFR1 and RIP5, was noticed within the collecting ducts with the predominant expression of FGFR1 (Figure 1a).

The percentage of FGFR1-positive cells differed significantly between E13.5 and E15.5 in both animal genotypes (Figure 1f and Figure S3a). A significant difference was noticed in all observed structures at E15.5, where *yotari* exhibited a higher percentage of FGFR1-positive cells than wt mice (Figure 1f and Figure S3a). FGFR1 was moderately expressed in the epithelial cells of the ureter and nearby concentric layers of ureteric wall muscles, while expression decreased towards peripheral connective tissue. As for the semi-quantitative analysis, wt mice at E15.5 had shown moderate staining intensity of FGFR1 in the collecting ducts and mild reactivity in the rest of the structures. *Yotari* mice expressed different intensity patterns, where convoluted tubules and renal vesicle stages, more mature forms of nephrons, had shown moderate and collecting duct mild FGFR1 reactivity (Table 1). Merging the images of the two markers, FGFR1 and RIP5, disclosed their co-expression in the parietal epithelial cells of immature glomeruli (Figure 1c,d) and at the marginal zone between developing nephrons and metanephric mesenchyme (Figure 1b and Figure S2b).

**Figure 2:**
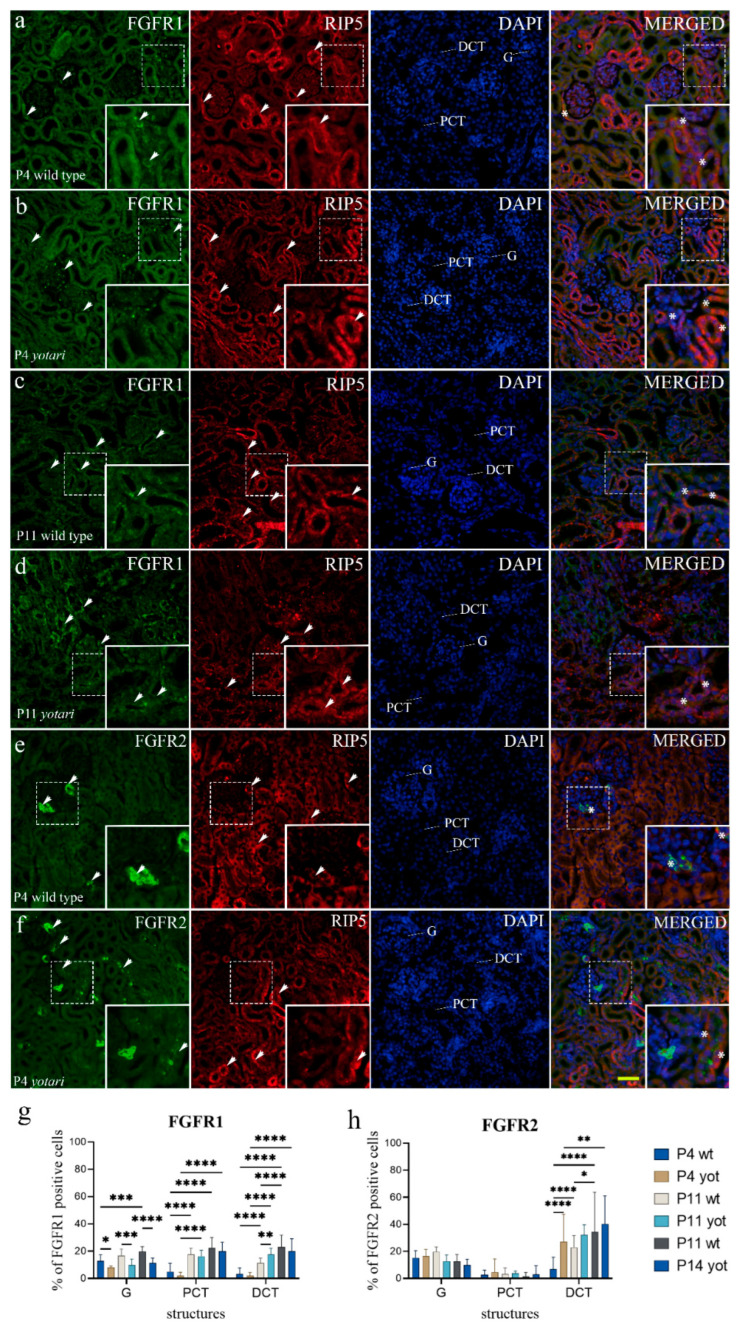
Double immunofluorescence staining of postnatal wild-type and *yotari* mouse kidneys with the FGFR1 (**a**–**d**), FGFR2 (**e**,**f**), and RIP5 markers (**a**–**f**). Arrows show the expression pattern of FGFR1, FGFR2, and RIP5 in glomeruli (G), proximal convoluted tubules (PCT), and distal convoluted tubules (DCT) indicated on 4′,6-diamidino-2-phenylindole (DAPI) image. Immunoexpression of FGFR1, RIP5, DAPI staining and merged FGFR1, RIP5, and DAPI at 4 days (P4) and 11 days (P11) in wild type (**a**,**c**) and *yotari* (**b**,**d**). Wild-type and *yotari* mice at postnatal days P11 and P14 of kidney development mostly corresponded to the localization and intensity of FGFR1; thus, representative images were selected from P11. Immunoexpression of FGFR2, RIP5, DAPI staining and merged FGFR2, RIP5, and DAPI at P4 in wild type (**e**) and *yotari* (**f**). Wild-type and *yotari* mice at P4, P11, and P14 of kidney development mostly corresponded regarding localization and intensity of FGFR2; thus, representative images were selected from P4. The most prominent protein expression area is shown in inserts, corresponding to the dashed boxes. An asterisk denotes the zone where co-expression was detected. Images were taken on magnification ×40. The scale bar is 50 μm, which refers to all images. The distribution of the percentages of FGFR1 (**g**) and FGFR2 (**h**) positive cells in the glomeruli (G), proximal convoluted tubules (PCT), and distal convoluted tubules (DCT) of postnatal kidneys of wild type and *yotari* animals over time (P4, P11, P14). Data are presented as the mean ± SD (vertical line) and analyzed by a two-way ANOVA test followed by Tukey’s multiple comparison test. Significant differences were indicated by * *p* < 0.05, ** *p* < 0.001, *** *p* < 0.0001, **** *p* < 0.00001. At each time point, twenty substructures were assessed.

Concerning postnatal day P4, the percentage of FGFR1-positive cells decreased to less than 20% in the glomeruli of all examined animals (Figure 2g and Figure S4a). The expression levels in both PCT and DCT matched the expression level of convoluted tubules at E13.5 and E15.5. (Figure 2g and Figure S4a). The FGFR1 staining was perinuclear in the glomerular cells, whereas, in the PCT and DCT, it was punctate and dispersed throughout the cytoplasm (Figure 2a,b and Figure S2c,d).

The percentage of FGFR1-positive cells within all observed structures increased through time (*p* < 0.05, Figure 2g and Figure S4a). At P4, P11, and P14, the percentage of positive cells was significantly higher in the glomeruli of wt animals than *yotari* animals (*p* < 0.05, Figure S4a). At P11, *yotari* demonstrated a significant increase in FGFR1-positive cells percentage in DCT, compared to wt (*p* < 0.05, Figure 2g and Figure S4a).

Semi-quantitative analysis revealed mild to moderate reactivity in glomeruli and mild staining intensity in all other observed structures of wt and *yotari* mice at P4. The staining intensity increased over time, with moderate at P11, which in PCT and DCT at P14 was leaning towards strong staining reactivity (Table 2). Merging the expression of two markers, FGFR2 and RIP5, disclosed their co-expression at DCT with the predominant expression of RIP5 at all observed postnatal days (Figure 2b–d).

### 2.2. FGFR2 Expression

**Figure 3:**
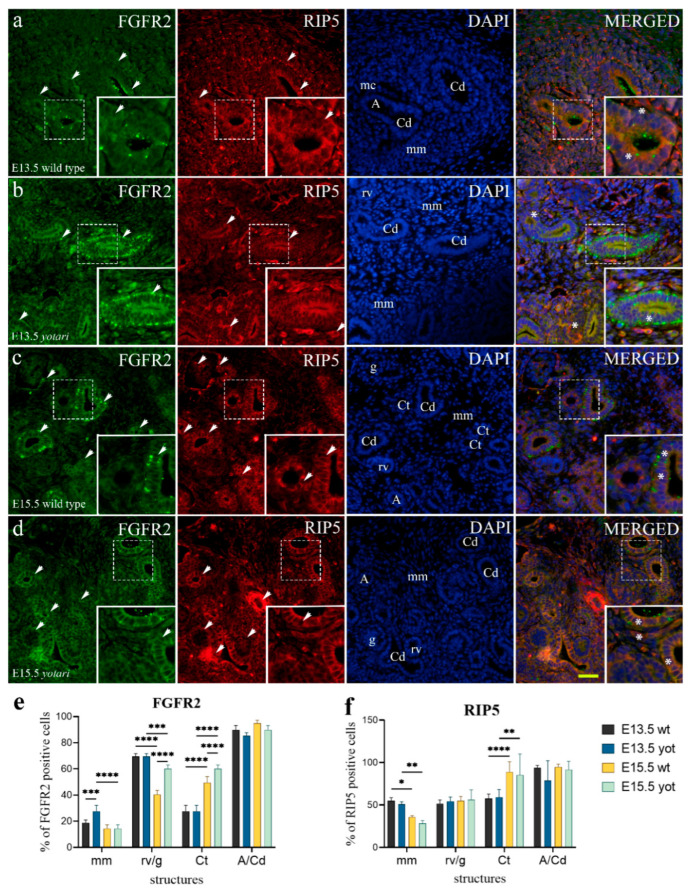
Double immunofluorescence staining of embryonic wild-type and *yotari* mouse kidneys with the FGFR2 and RIP5 markers (**a**–**d**). Arrows show the expression pattern of FGFR2 and RIP5 in metanephric mesenchyme (mm), renal vesicles (rv), glomeruli (g), convoluted tubules (Ct), ampullae (A), and collecting ducts (Cd) indicated on 4′,6-diamidino-2-phenylindole (DAPI) image. Immunoexpression of FGFR2, RIP5, 4′,6-diamidino-2-phenylindole (DAPI) staining and merged FGFR2, RIP5, and DAPI at embryonic days E13.5 and E15.5 in wild type (**a**,**c**) and *yotari* (**b**,**d**). The most prominent protein expression area is shown in inserts, corresponding to the dashed boxes. An asterisk denotes the zone where co-expression was detected. Images were taken on magnification ×40. The scale bar is 50 μm, which refers to all images. The distribution of the percentages of FGFR2 (**e**), and RIP5 (**f**) positive cells in the metanephric mesenchyme (mm), renal vesicles (rv) or glomeruli (g), convoluted tubules (Ct), and ampulla (A) or collecting ducts (Cd) of wild type and *yotari* kidneys at embryonic days E13.5 and E15.5. Data are presented as the mean ± SD (vertical line) and analyzed by a two-way ANOVA test followed by Tukey’s multiple comparison test. Significant differences were indicated by * *p* < 0.05, ** *p* < 0.001, *** *p* < 0.0001, **** *p* < 0.00001. At each time point, ten substructures were assessed.

FGFR2 shows a similar expression pattern to FGFR1 in the identical kidney substructures of both animal genotypes: an abundance of strong punctate staining of the apical epithelial membrane of collecting ducts which sometimes occurs within the basolateral membrane, and developing nephrons within the nephrogenic zone, and the moderate expression in convoluted tubules and metanephric mesenchyme at E13.5 (Figure 3a,b). A significant difference was observed in undifferentiated mesenchyme, where *yotari* exhibited higher expression proportions compared to wt mice (Figure 3e and Figure S3b).

Semi-quantitative analysis of *yotari* mice revealed mild staining intensity within all observed structures, whereas mitotic cells of renal vesicle stages and collecting ducts of wt mice exhibit moderate reactivity (Table 1).

The percentage of FGFR2-positive cells significantly differed between E13.5 and E15.5 in both animal genotypes. A significant difference was observed in immature glomeruli and convoluted tubules at E15.5, where *yotari* exhibited a higher percentage of FGFR2-positive cells than wt mice Figure 3e and Figure S3b).

Merging the two markers FGFR2 and RIP5 revealed their co-expression in the parietal epithelial cells of immature glomeruli and within collecting ducts with predominant FGFR2 expression (Figure 3c,d). Punctate RIP5 staining was seen at apical cell-to-cell junctions lining the ureteric bud epithelia (Figure 3b,c).

On postnatal day P4, the percentage of FGFR2-positive cells showed a gradual reduction in all observed structures compared to the expression rate of renal vesicle stages, immature glomeruli, and convoluted tubules at E13.5 and E15.5 of both *yotari* and wt animals (Figure 2h and Figure S4b). In the analysis of postnatal kidney structures, strong punctate FGFR2 staining was observed in the apical membrane of nephron tubules, specifically DCT accompanied by intense diffuse cytoplasmatic staining in juxtaglomerular apparatus (JGA) and endothelial cells of blood vessels (Figure 2e,f). In the G and PCT, FGFR2 expression appeared constant regardless of postnatal age and genotype (Figure 2h and Figure S4b). On the other hand, a significant increase in FGFR2 immunoexpression in the DCT was found in more advanced developmental stages P11 and P14 in wt and P14 in *yotari*, respectively (*p* < 0.05, Figure 2h and Figure S4b). Only in the DCT of the P4 *yotari* mice was the percentage of positive cells significantly higher than in control animals (*p* < 0.01, Figure 2h and Figure S4b). As for the semi-quantitative analysis, wt mice at day P4 showed vigorous staining intensity in glomeruli and JGA and mild in tubules, while P11 and P14 displayed strong reactivity in both JGA and DCT but mild in PCT. Regardless of age, *yotari* tubules were mild to moderately positive, but the glomeruli were stronger in intensity at P14 (Table 2).

Merging the expression of the two markers, FGFR2 and RIP5, disclosed their co-expression at JGA with the predominant expression of FGFR2 (Figure 2e). The co-expression was noticed in the walls of blood vessels and distal tubular cells with the predominant expression of RIP5 at all observed postnatal days (Figure 2f).

### 2.3. RIP5 Expression

RIP5 positive cells were recognized as red staining in the basolateral and apical membranes (Figure 1a and Figure 3a). At E13.5 of kidney cortex development, RIP5 was moderately expressed in the undifferentiated cells of metanephric mesenchyme (interstitium), developing nephrons (metanephric cup, renal vesicle stages) and walls of blood vessels, but strongly in the epithelium of collecting ducts, including ampullae (Figure 1a,b and Figure S2a,b). At E15.5, nephrogenesis advanced at the expense of undifferentiated metanephric mesenchyme with the consequence of lower RIP5 expression, accompanied by strong RIP5 positivity in the walls of intra-glomerular and extra-glomerular blood vessels (Figure 3c,d). Additionally, we found a more prominent expression pattern in immature glomeruli, specifically in the parietal layer of the Bowman’s capsule opposed to expression in renal vesicle stages (Figure 1c). The RIP5 expression rate did not display any significant difference between the embryonic developmental phases of wt and *yotari* (Figure 3f and Figure S3c). Semi-quantitative analysis of wt on E13.5 revealed strong staining intensity in metanephric mesenchyme and convoluted tubules, mild reactivity in mitotic cells of renal vesicle convoluted tubules, and moderate in collecting ducts. Same age *yotari* specimens displayed moderate staining intensity within all observed structures. At E15.5, both animal genotypes displayed similar expression reactivity: mild in mm, moderate in remaining substructures which in immature glomeruli of wt animals was sometimes inclining towards strong (Table 1).

**Figure 4:**
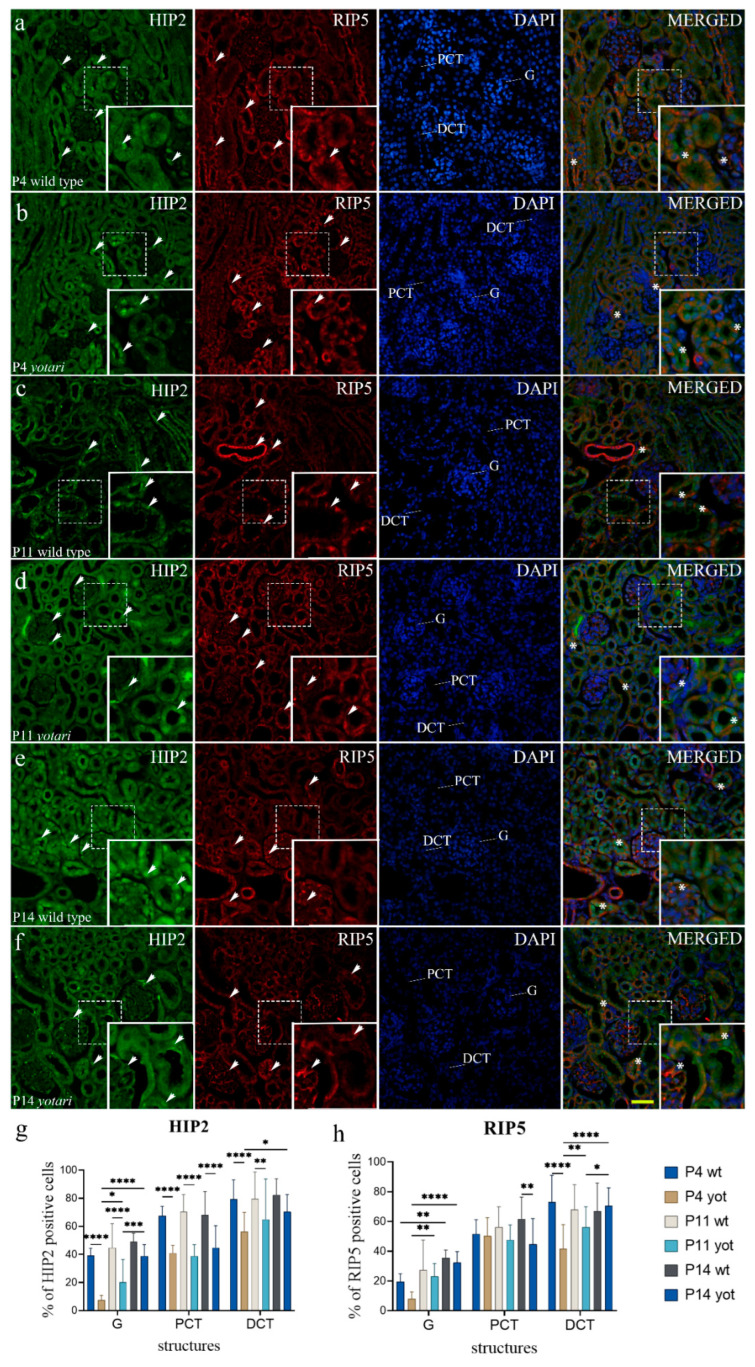
Double immunofluorescence staining of postnatal wild-type and *yotari* mice kidneys with the HIP2 and RIP5 markers (**a**–**f**). Arrows indicate the expression pattern of HIP2 and RIP5 in glomeruli (G), proximal convoluted tubules (PCT), distal convoluted tubules (DCT). Immunoexpression of HIP2, RIP5, 4′,6-diamidino-2-phenylindole (DAPI) staining and merged HIP2, RIP5, and DAPI at postnatal days 4 (P4), 11 (P11) and 14 (P14) in the wild type (**a**,**c**,**e**) and *yotari* (**b**,**d**,**f**). The most prominent protein expression area is shown in inserts, corresponding to the dashed boxes. An asterisk denotes the zone where co-expression was detected. Images were taken on magnification ×40. The scale bar is 50 μm, which refers to all images. The distribution of the percentages of HIP2 (**g**) and RIP5 (**h**) positive cells in the glomeruli (G), proximal convoluted tubules (PCT), and distal convoluted tubules (DCT) of postnatal kidneys of wild type and *yotari* animals over time (P4, P11, P14). Data are presented as the mean ± SD (vertical line) and analyzed by a two-way ANOVA test followed by Tukey’s multiple comparison test. Significant differences were indicated by * *p* < 0.05, ** *p* < 0.001, *** *p* < 0.0001, **** *p* < 0.00001. At each time point, twenty substructures were assessed.

In the cortex of wt mice kidneys (P4), strong expression of RIP5 was noticed in the apical cytoplasm of proximal and distal tubules (Figure 2a) and endothelial walls of blood vessels (Figure 4a), but mild in glomeruli, with noticeable staining in its parietal epithelial cells (Figure 2a). On the P14, cortical expression of RIP5 increased significantly in PCT and DCT of the wt animals and in G and PCT of the *yotari* animals (*p* < 0.05, Figure 4h).

The expression pattern in G at P14 differed from that of P4, with an increased expression rate in the glomerular cell population (Figure 4e,f). Analysis revealed significant differences between the two genotypes: the RIP5 expression in DCT was vaster at P4 wt than in *yotari* (*p* < 0.05, Figure 4h and Figure S4c). The expression in PCT also increased on P14 (*p* < 0.05, Figure 4h and Figure S4c). In the semi-quantitative evaluation, wt mice revealed mild to moderate staining intensity in the G and PCT and strong staining intensity in the DCT at P4. Same age *yotari* specimens displayed milder intensity in every structure type except for the PCT, where intensity increased. Cortical tubules and glomeruli of wt and *yotari* mice of P11 age showed moderate reactivity. Lastly, the PCT of P14 wt and *yotari* mice were moderately stained, while the glomeruli and DCT were noticeably stronger in intensity (Table 2).

As previously described, RIP5 co-expressed with FGFR1 and FGFR2 in both embryonic and postnatal kidney substructures. The co-expression of RIP5 and HIP2 markers is described below.

### 2.4. HIP2 Expression

In the analysis of HIP2, fluorescence appeared constant regardless of embryonic stages and genotype. Cytoplasmatic staining was found in punctate and diffuse forms (Figure 1e,f). At the embryonic days E13.5 and E15.5, HIP2 was strongly expressed in collecting ducts and convoluted tubules and moderately in developing nephrons (metanephric cup, renal vesicle) (Figure 1g and Figure S3d). The HIP2 expression rate did not display any significant difference between the embryonic developmental phases of wt and *yotari* (Figure 1g and Figure S3d). Concerning intensity of immunoreactivity, both animal genotypes demonstrated a similar pattern: mild reactivity in mm and rv/g and moderate in Ct and A/Cd at E13.5, in the later phase inclining towards strong values (Table 1). Co-expression of RIP5 and HIP2 markers was found in almost all of the observed substructures: collecting ducts with predominant expression of RIP5 (Figure 1e) and renal vesicle stages with the predominant expression of HIP2. The areas of only HIP2 expression were occasionally seen due to the difference in the spatial expression of RIP5 and HIP2 (Figure 1e).

HIP2 positive cells displayed strong punctate staining at the postnatal period, with mainly all positive cells in a structure in wt and *yotari* mice (Figure 4e). Positive cells were observed in the parietal epithelial cells of glomeruli, apical membrane of PCTs, and DCTs, with the signal being localized mainly within the nucleus. In the more advanced developmental phases, the staining in the capsular space of the *yotari* mice glomeruli was noticed (Figure 4d). The rate of HIP2 positive cells within all observed structures of both animal genotypes increased through time (Figure 4h and Figure S4d). Significant differences in HIP2 expression rate were found in all control groups and the *yotari* (*p* < 0.05, Figure 4h and Figure S4d). The expression in the glomeruli of wt animals was far vaster at P14 than P4. *Yotari* demonstrated a significant increase in expression rate in glomeruli at all observed time points and in distal convoluted tubules at P14, compared to P4 (*p* < 0.05, Figure 4h and Figure S4d). The analysis between the observed structures of wt and *yotari* animals revealed a discernible difference at P4 and P11 (*p* < 0.05, Figure 4h and Figure S4d). A decrease in the HIP2 expression rate at P14 was noted in PCTs of *yotari*, compared to wt mice (*p* < 0.05, Figure 4h and Figure S4d). The intensity of tubular staining at P4 was weak, while the glomeruli, regardless of genotype, demonstrated a moderate fluorescence signal (Table 2). In the more advanced developmental phases, signal intensity was moderate to strong (Table 2). Merging the two markers, HIP2 and RIP5, disclosed their co-expression at glomeruli and DCTs (Figure 4a,b,d).

### 2.5. Erk1/2 and mTOR Expression

**Figure 5:**
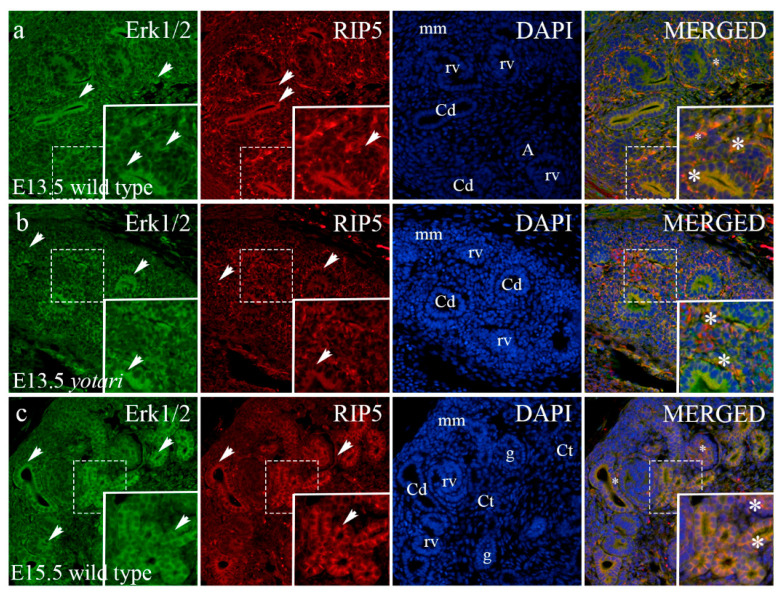

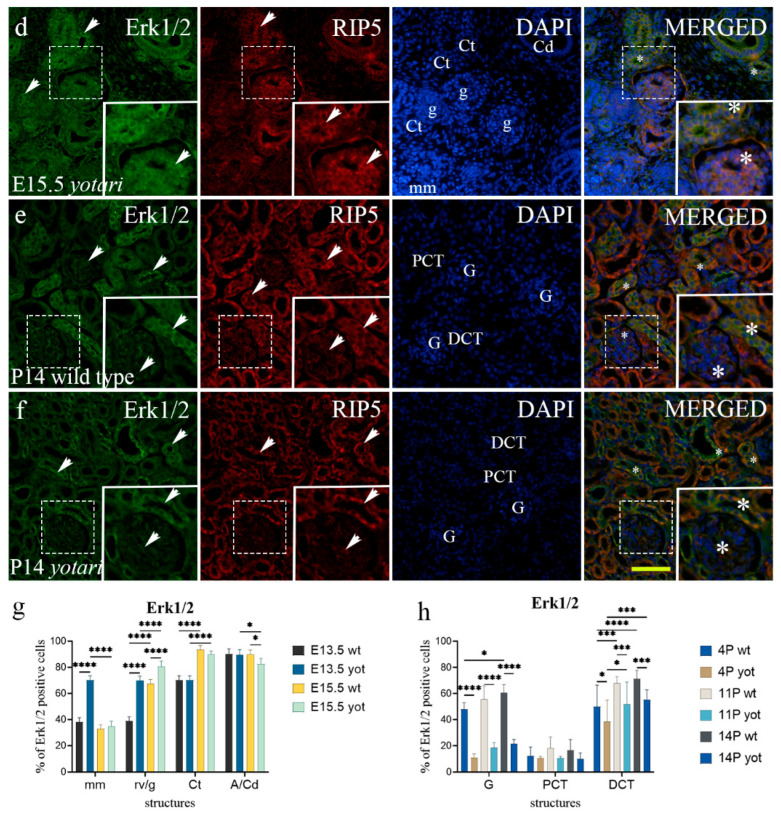
Double immunofluorescence staining of embryonic wild-type (**a**,**c**) and *yotari* (**b**,**d**) and postnatal wild-type (**e**) and *yotari* (**f**) mouse kidneys with the Erk1/2 and RIP5 markers. Arrows show the expression pattern of Erk1/2 and RIP5 in the mesenchyme (mm), renal vesicles (rv), immature glomeruli (g), convoluted tubules (Ct), ampullae (A), and collecting ducts (Cd), glomeruli (G), proximal convoluted tubules (PCT) and distal convoluted tubules (DCT) indicated on the 4′,6-diamidino-2-phenylindole (DAPI) image. Immunoexpression of Erk1/2, RIP5, DAPI staining and merged Erk1/2, RIP5, and DAPI at embryonic days E13.5 and E15.5 in wild type (**a**,**c**) and *yotari* (**b**,**d**). Immunoexpression of Erk1/2, RIP5, DAPI staining and merged Erk1/2, RIP5, and DAPI at postnatal day 14 (P14) in wild type (**e**) and *yotari* (**f**). Wild-type and *yotari* mice at postnatal day 4 (P4) and 11 (P11) of kidney development mostly correspond regarding localization and intensity of Erk1/2; thus, a representative image was selected from P14. The most prominent protein expression area is shown in inserts, corresponding to the dashed boxes. An asterisk denotes the zone where co-expression was detected. Images were taken on magnification ×40. The scale bar is 50 μm, which refers to all images. The distribution of the percentages of Erk1/2 positive cells in the metanephric mesenchyme (mm), renal vesicles (rv) or glomeruli (g), convoluted tubules (Ct), and ampulla (A) or collecting ducts (Cd) of wild type and *yotari* kidneys at embryonic days E13.5 and E15.5 (**g**). The distribution of the percentages of Erk1/2 positive cells in the glomeruli (G), proximal convoluted tubules (PCT), and distal convoluted tubules (DCT) of postnatal kidneys of wild type and *yotari* animals over time (P4, P11, P14) (**h**). Data are presented as the mean ± SD (vertical line) and analyzed by a two-way ANOVA test followed by Tukey’s multiple comparison test. Significant differences were indicated by * *p* < 0.05, *** *p* < 0.0001, **** *p* < 0.00001. At each embryonic time point, ten substructures were assessed. At each postnatal time point, twenty substructures were assessed.

The percentage of Erk1/2-positive cells significantly differed at E13.5 and E15.5 between the two animal genotypes. A significant increase in Erk1/2 expression was noticed at E13.5 in the renal vesical stage and metanephric mesenchyme of *yotari* animals (*p* < 0.05; Figure 5g and Figure S5b). At E15.5, the level of Erk1/2-positive cells significantly increased in immature glomeruli and decreased in collecting ducts of *yotari* mice (Figure 5g and Figure S5b). Merging the two markers Erk1/2 and RIP5 revealed their co-expression in the parietal epithelial cells of immature glomeruli and within convoluted tubules (Figure 5a,c,d). Concerning postnatal developmental stages, the *yotari* animals showed a significant decrease in the Erk1/2 expression within all observed structures at all observed time points (*p* < 0.05, Figure 5h and Figure S5d). The co-expression was noticed in the glomeruli and distal convoluted tubules (Figure 5e,f).

**Figure 6:**
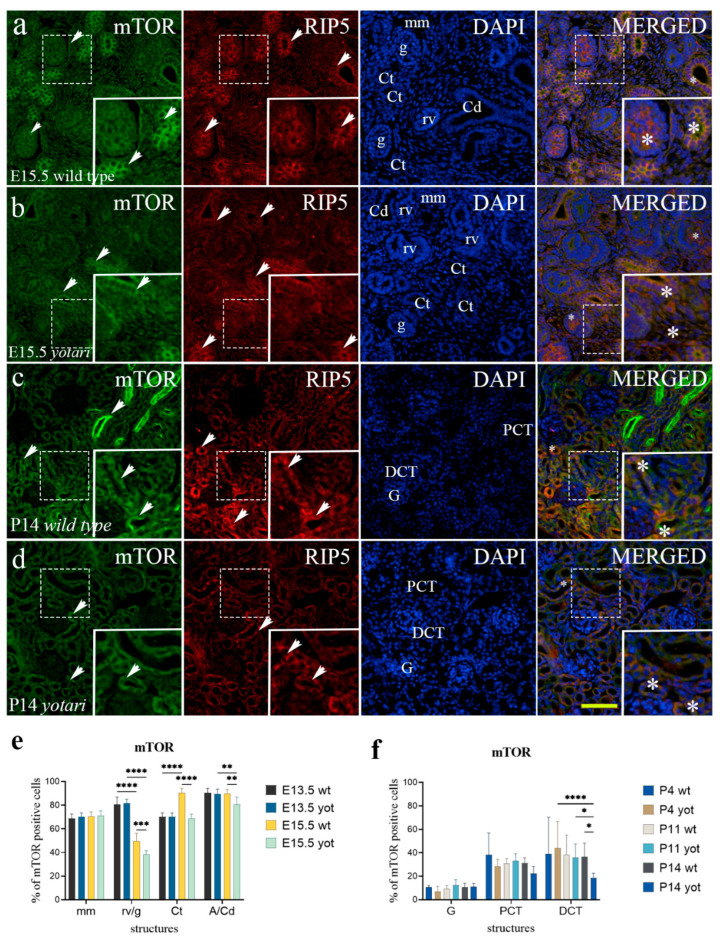
Double immunofluorescence staining of embryonic wild-type (**a**) and *yotari* (**b**) and postnatal wild-type (**c**) and *yotari* (**d**) mouse kidneys with the mTOR and RIP5 markers. Arrows show the expression pattern of mTOR and RIP5 in the mesenchyme (mm), renal vesicles (rv), immature glomeruli (g), convoluted tubules (Ct), ampullae (A), and collecting ducts (Cd), glomeruli (G), proximal convoluted tubules (PCT) and distal convoluted tubules (DCT) indicated on 4′,6-diamidino-2-phenylindole (DAPI) image. Immunoexpression of mTOR, RIP5, DAPI staining and merged mTOR, RIP5, and DAPI at embryonic day E15.5 in wild type (**a**) and *yotari* (**b**). Immunoexpression of mTOR, RIP5, DAPI staining and merged mTOR, RIP5, and DAPI at postnatal day 14 (P14) in wild type (**c**) and *yotari* (**d**). Wild-type and *yotari* mice at embryonic day E13.5 of kidney development mostly correspond regarding localization and intensity of mTOR; thus, a representative image was selected from E15.5. Wild-type and *yotari* mice at postnatal day 4 (P4) and 11 (P11) of kidney development mostly correspond regarding localization and intensity of mTOR; thus, a representative image was selected from P14. The most prominent protein expression area is shown in inserts, corresponding to the dashed boxes. An asterisk denotes the zone where co-expression was detected. Images were taken on magnification ×40. The scale bar is 50 μm, which refers to all images. The distribution of the percentages of mTOR positive cells in the metanephric mesenchyme (mm), renal vesicles (rv) or glomeruli (g), convoluted tubules (Ct), and ampulla (A) or collecting ducts (Cd) of wild type and *yotari* kidneys at embryonic days E13.5 and E15.5 (**e**). The distribution of the percentages of mTOR positive cells in the glomeruli (G), proximal convoluted tubules (PCT), and distal convoluted tubules (DCT) of postnatal kidneys of wild type and *yotari* animals over time (P4, P11, P14) (**f**). Data are presented as the mean ± SD (vertical line) and analyzed by a two-way ANOVA test followed by Tukey’s multiple comparison test. Significant differences were indicated by * *p* < 0.05, ** *p* < 0.001, *** *p* < 0.0001, **** *p* < 0.00001. At each embryonic time point, ten substructures were assessed. At each postnatal time point, twenty substructures were assessed.

Concerning mTOR expression, the only significant difference in embryonic stages was found at E15.5, where *yotari* exhibited a decrease in almost all observed structures except in metanephric mesenchyme, where we noticed no difference between the groups (Figure 6e and Figure S5a). Merging the two markers, mTOR and RIP5, disclosed their co-expression at convoluted tubules and immature glomeruli (Figure 6a,b). In the postnatal developmental phases analysis, the only significant difference was observed in distal convoluted tubules where mTOR expression levels decreased in *yotari* (Figure 6f and Figure S5c). An intriguing pattern of mTOR expression was detected in the medullary rays of the kidney cortex. In these structures, mTOR is expressed in a more robust pattern accompanied by stronger fluorescence intensity (Figure 6c). mTOR and RIP5 co-expressed in proximal and distal convoluted tubules (Figure 6c,d).

## 3. Discussion

Kidney morphogenesis, development, and maturation are intricate processes precisely coordinated through the interplay of many genes. Using a *Dab1^−/−^* mice model, we characterized the significant differences in spatio-temporal distribution patterns of FGFR1, FGFR2, RIP5, and HIP2, in both embryonic and postnatal developmental stages. The fact that the expression of the aforementioned proteins was detected in all investigated phases of kidney development [13,15,20,26,37,38] implies their critical role in early metanephric mesenchymal patterning, ureteric bud branching morphogenesis, nephrogenesis, and nephron progenitor survival. As FGFR1, FGFR2, RIP5, and HIP2 proteins are expressed in all parts of the nephron and some in the renal vasculature, it is justifiable to speculate about their significance in the maintenance of overall homeostasis and the maturation of kidney structures, as well as their contribution in the occurrence of different kidney pathologies.

Over the last couple of years, some genetic mutations responsible for structural kidney disease have been explored, including mutations in FGFRs [39,40,41,42]. As FGFR activation causes a pro-proliferative, anti-apoptotic, and pro-survival response [20,22,25], it is justifiable to speculate about the impaired cell response caused by *Dab1* silencing. Our prior research revealed the CAKUT phenotype resulting in *yotari* [2]. Since then, we have proposed various mechanisms that may influence the *yotari* mice’s kidney and liver structures, including glomeruli, blood vessels, and the tubulointerstitial tissue [3,43].

This study showed a significantly reduced percentage of FGFR1 positive cells in the glomeruli of *yotari* mice at all observed postnatal time points. Diminishing FGFR1 expression in the glomeruli of *yotari* mice may potentially contribute to the previously mentioned occurrence of renal hypoplasia and foot process effacement, but the exact mechanism still needs to be clarified. Expression of the FGFR1 during embryonic development was the highest in the rv/g and A/Cd, suggesting the importance of this protein in epithelial patterning rather than involvement in mesenchymal to epithelial transformation.

FGFR2 immunoexpression was observed in immature glomeruli and convoluted tubules at gestation, where *yotari* exhibits elevated expression proportions compared to wild-type mice. In the postnatal period, only in the DCT of the P4 *yotari* mice was the percentage of positive cells significantly higher than control animals. These results point to the essential role of FGFR2 in early embryonic metanephric patterning and nephrogenesis, while its importance in the maintenance of kidney function is diminished.

Although there are currently no studies comparing the immunoexpression of Dab1, Erk1/2, and FGFR proteins in the kidney, in the study of Kon et al., it has been shown that Reelin, an extracellular matrix glycoprotein upstream of Dab1, regulates the neuronal positioning, preventing FGFR degradation through Erk1/2 phosphorylation. Moreover, results suggest that Reelin-induced Erk1/2 phosphorylation is dependent on FGFR activity and correlates with its effect on FGFR protein levels [44].

Studies in primary neurons demonstrated that activation of Reelin’s downstream receptor apolipoprotein E (apoE) resulted in tyrosine phosphorylation of Dab1 and activation of Erk1/2, whereas experiments involving *Dab1* knockout neurons revealed that Dab1 was not essential for Erk activation [45]. Another study on cortical neurons has shown that Dab1 phosphorylation promotes subsequent Src family kinase (SFK) activation in a positive feedback loop, which may explain why it is partially implicated in Reelin-mediated MEK/Erk1/2 activation. The same study examined Dab1 deficiency and potential signaling abnormalities in vivo, analyzing the forebrains of wild-type and heterozygous Dab1 knockout mice. According to the findings, the basal phosphorylation levels of Akt and Erk1/2 were significantly reduced in juvenile (3–4 weeks old) heterozygous Dab1 knockout mice compared to wild type [46]. Similarly, in our study, both Erk1/2 and mTOR, a downstream effector of Akt, expression in *yotari* kidneys decreased in embryonic phases. Concerning postnatal developmental stages, the *yotari* animals showed a significant decrease in the Erk1/2 expression within all observed structures at all observed time points. Therefore, we can hypothesize that a similar signaling pathway model exists in kidneys: the Erk1/2 signaling pathway in the *yotari* mice kidneys is dependent on Reelin with Dab1 only partially implicated in Reelin-mediated MEK/Erk1/2 activation.

The significant difference at embryonic stages in the percentage of mTOR-positive cells was found at E15.5, where *yotari* exhibited a decrease in almost all observed structures except in metanephric mesenchyme. In the postnatal developmental phases analysis, a significant decrease in mTOR cell immunoexpression was observed in distal convoluted tubules of *yotari* mice kidneys. In the study of Jossin et al., it is demonstrated that Reelin activates the mTOR-S6K1 (S6 kinase 1) pathway, dependent on Dab1 phosphorylation by Src kinases and activation of PI3K/Akt/mTOR. The study concluded that PI3K and Akt are necessary for cortical development control and mTOR for dendritic growth and branching regulation [47]. Therefore, we can assume that *Dab1* inactivation in the kidneys of *yotari* mice inevitably results in a reduced mTOR expression level.

Our previous studies on human kidneys reported that the cells of undifferentiated metanephric mesenchyme predominantly express RIP5 [13]. In the study of Sanna-Cherchi et al. [11], it has been shown that RIP5 has a striking membrane-associated distribution in mesenchymal-derived cells of all major organs. In addition, the developing mouse kidney expresses RIP5 at low levels in the nephrogenic zone but more highly in maturing tubular epithelia. In our study, similar results were obtained: RIP5 positive cells were recognized as red staining in the basolateral and apical membranes of wild-type and *yotari* animals and seen at apical membrane lining the ureteric bud epithelia. The moderate protein expression was detected in the metanephric mesenchyme cells, developing nephrons and blood vessels’ walls but it was strong in the epithelium of collecting ducts, including ampullae. Merging the two markers FGFR2 and RIP5 revealed their co-expression in the parietal epithelial cells of immature glomeruli and, together with FGFR1, within the ureteric bud. The findings from this and our recent study on human kidneys [13] suggest the great importance of RIP5 in the earliest induction stages of nephrogenesis and maturation and, together with FGFR1 and FGFR2 in the vasculogenesis due to markers’ co-expression in the vascular walls. It has been demonstrated that RIP5 knockdown in human embryonic kidney cells inhibited FGF-stimulated phosphorylation of Erk, which is the main effector of FGF-induced transcriptional activity. These data, combined with the observed co-expression of RIP5 with FGFR1 and FGFR2, implicate RIP5 in the downstream regulation of FGF signaling [11]. RIP5′s biological role, on the other hand, is still largely unclear. The study has shown that overexpression of RIP5 induces cell death associated with caspase-dependent and caspase-independent apoptotic pathways [48]. In addition, an increased level of apoptosis was observed in the glomeruli of P14 *yotari* mice due to elevated cleaved Casp-3 expression [2]. The analyzed structures of *yotari* samples exhibited insignificant fluctuation of RIP5 immunoreactivity between postnatal phases of development. The only significant difference was observed in DCT at P4 and PCT at P14 due to decreased RIP5 expression in *yotari*. Therefore, these findings cannot tie together the link between RIP5 expression and the expected level of apoptosis in cell responses to silenced *Dab1* in *yotari* mice, suggesting that the process of apoptosis is triggered via other signaling pathways.

This research is the first instance in which HIP2 has been investigated in developing mice kidneys. Our current study, and the recent one [13], confirmed its expression in the mice and human kidneys. Our results demonstrated the strong HIP2 expression in collecting ducts and convoluted tubules and moderate expression in developing nephrons (metanephric cup, renal vesicle). During the postnatal period, positive cells were observed in the parietal epithelial cells of glomeruli, apical membrane of proximal, and distal convoluted tubules. Co-expression of RIP5 and HIP2 markers has been noticed in almost all observed substructures: collecting ducts and renal vesicle stages at gestation and later, postnatally, in glomeruli and distal convoluted tubules. Microphotograph observations showed that the protein did not discriminate between the observed substructures at embryonic developmental phases of wt and *yotari*. However, the analysis revealed a discernible difference during the postnatal period, where we noted a significant decrease in the HIP2 expression in almost all of the observed structures of *yotari* animals. Previous studies show that decreasing HIP2 expression may increase the risk of dopaminergic neuronal death and motor function impairment [49] and suppress the cell cycle, cell proliferation, cell migration, and wound healing in gastric cancer cells [50]. Therefore, our findings imply the critical importance of HIP2 in suppressing cell death during nephrogenesis and maturation in wild-type mice. As the aforementioned studies emphasize the consequences of decreased HIP2 immunoexpression, such as cell apoptosis induction and wound healing suppression, it is reasonable to speculate about a possible connection between decreased HIP2 immunoexpression in postnatal kidney structures and podocyte injury followed by *yotari* mice’s premature death.

A shortcoming of our current study is the inability to show the interactions of FGFR1 with HIP2 and FGFR2 with HIP2. Furthermore, we did not perform Western blots or validate antibodies in cell cultures to determine their specificity. Another interesting point that we did not consider is the FGFR1 expression and its impact on the receptor of inositol-1-4,5 trisphosphate (IP_3_). This knowledge would be important in further elucidating and broadening the context of *yotari* mouse genesis.

To summarize, the abundant presence of observed proteins in kidneys and the dynamics of their expression found in this study suggest that FGFR1, FGFR2, RIP5, and HIP2 play essential roles not only in early metanephric mesenchymal patterning, ureteric bud branching morphogenesis, nephrogenesis, and nephron progenitor survival but also in the maintenance of overall homeostasis and the maturation of kidney structures in the postnatal phase. The effect of *Dab1^−/−^* functional silencing on kidney nephrogenesis and function is confirmed by statistically significant variations in the spatio-temporal expression patterns of the investigated markers between *yotari* and wild-type mice. Still, the exact mechanism through which the Reelin/Dab1 pathway influences the expression of examined markers remains to be elucidated. The decrease in Erk1/2 and mTOR expression in *yotari* kidneys can be explained by a proposed signaling pathway in which Dab1 is only partially implicated in Reelin-mediated MEK/Erk1/2 activation. Our findings underscore the critical role of the investigated markers throughout normal kidney development and their potential involvement in kidney pathology and diagnostics, where they might serve as biomarkers and therapeutic targets.

## 4. Materials and Methods

### 4.1. Ethics

Animal use was approved by the Guidelines for the Care and Use of Laboratory Animals at the Shiga University of Medical Science. The study was conducted according to the guidelines of the Declaration of Helsinki and approved by the Ethical Committee of the University of Split School of Medicine (UP/1-322-01/17-01/13; 525-10/0255-17-7; 13 October 2017).

### 4.2. Generation of Dab1 Null Conventional Mutants and Sample Collection

This experiment used *yotari* (*Dab1^−/−^*) mice as *Dab1* null conventional mutants, constructed as previously described [1,6,7]. C57BL/6N mice were raised and group-housed in standard polycarbonate cages (3–4 animals, including at least one of each genotype) with free access to food and tap water in a temperature-controlled (23 + 2 °C) room. The photoperiod consisted of 12 h of artificial light and 12 h of darkness. The following PCR primers were used for genotyping: *yotari*—GCCCTTCAG-CATCACCATGCT and CAGTGAGTACATATTGTGTGAGTTCC, wild-type of *Dab1* locus—GCCCTTCAGCATCACCATGCT and CCTTGTTTCTTTGCTTTAAGGCTGT [51]. The gravid mice were sacrificed on gestation days 13.5 (E13.5) and 15.5 (E15.5), and their embryos were obtained. Other groups of mice were sacrificed on the postnatal days 4, 11, and 14 (P4, P11, P14). Three to four animals were used per examined group. First, they were deeply anesthetized with pentobarbital and, afterward, transcardially perfused using phosphate buffer saline (PBS, pH 7.2) followed by 4% paraformaldehyde (PFA) in 0.1 M PBS. Kidneys were removed and separately fixed with 4% PFA in 0.1 M PBS overnight for conventional histological analyses (hematoxylin–eosin and immunofluorescence staining).

### 4.3. Immunofluorescence

Following fixation and dehydration of tissue with graded ethanol solutions, tissue was embedded in paraffin blocks and serially cut as five µm-thick sections, then mounted on coverslips. Proper tissue preservation was confirmed by hematoxylin–eosin staining of every 10th section. After deparaffinization in xylol and rehydration in graded water–ethanol solutions, the mounted tissue samples were heated in a water steamer in the 0.01 M citrate buffer (pH 6.0) for 30 min at 95 °C and then gradually cooled down to room temperature. After rinsing in 0.1 M PBS, protein blocking buffer (ab64226, Abcam, Cambridge, UK) was applied for 20 min to exclude nonspecific staining. Primary antibodies (Table 3) were applied on sections and incubated overnight in a humidity chamber. They were washed with PBS the next day before incubating with appropriate secondary antibodies (Table 3) for one hour. Lastly, the samples were washed in PBS once more, and DAPI (4′,6-diamidino-2-phenylindole) staining was used to visualize nuclei. Samples were air-dried and cover-slipped (Immuno-Mount, Thermo Shandon, Pittsburgh, PA, USA).

The preadsorption test was performed so that each primary antibody was diluted in blocking solution to the precisely determined concentration. A suitable peptide antigen was added and the combination applied to the sections. The results showed no antibody staining. No nonspecific binding of secondary antibodies or false-positive results was observed when primary antibodies were omitted from the immunofluorescence protocol.

### 4.4. Data Acquisition and Analysis

Sections were examined by immunofluorescence microscope (BX51, Olympus, Tokyo, Japan) equipped with a Nikon DS-Ri2 camera (Nikon Corporation, Tokyo, Japan). In order to quantify immunoexpression of proteins of interest, non-overlapping visual fields were captured at ×40 magnification, and constant exposure times were analyzed. We captured at least ten images of embryonic kidney substructures: metanephric mesenchyme (mm), renal vesicles (rv), immature glomeruli (g), convoluted tubules (Ct), ampullae (A), and collecting ducts (Cd) at embryonic days E13.5 and E15.5, and at least twenty images of postnatal kidney structures: glomeruli (G), proximal convoluted tubules (PCT), and distal convoluted tubules (DCT) at postnatal days P4, P11, and P14. All captured images were processed with ImageJ software (National Institutes of Health, Bethesda, MD, USA) and Adobe Photoshop (Adobe, San Jose, CA, USA). The number of RIP5, FGFR1, FGFR2, HIP2, Erk1/2, and mTOR immunoreactive cells were counted, expressed as a percentage of total cells, and averaged per animal group. Any level of cytoplasmic, nuclear, or membrane staining with used markers was regarded as positive. The staining intensity of distinct kidney structures was semi-quantitatively evaluated at four degrees: the absence of any reactivity (−), mild reactivity (+), moderate reactivity (++), and strong reactivity (+++) (Table 3). Considering inter-observer variations, three investigators analyzed the captured microphotographs independently. Interrater agreement was proved with interclass correlation analysis, which yielded a coefficient > 0.8, indicating excellent agreement [52].

### 4.5. Statistical Analyses

GraphPad Prism 8.0.1 software was used for statistical analyses (GraphPad Software, San Diego, CA, USA). A two-way ANOVA test followed by Tukey’s multiple comparison test was used to compare immunoexpression in order to determine significant differences in the percentage of positive cells between mm, rv/g, Ct, and A/Cd on E13.5 and E15.5 and G, PCT, and DCT at P4, P11, and P14. The percentage of positive cells was expressed as the mean + standard deviation (SD). The level of significance was set at *p* < 0.05.

## Figures and Tables

**Table 1:** Staining intensity of specific antibodies in the kidneys of *yotari* and wild-type mice at embryonic days E13.5 and E15.5.

Embryonic Day (E)	Animal	Structure	Antibody			
			FGFR1	FGFR2	RIP5	HIP2
E13.5	wild type	mm	+	+	+++	+
		rv/g	++	++	+	+
		Ct	+	+	+	++
		A/Cd	++	+++	++	++
	*yotari*	mm	+	+	++	+
		rv/g	++	+	++	+
		Ct	+	+	++	++
		A/Cd	++	+	++	++
E15.5	wild type	mm	+	+	+	+
		rv/g	+	++	+++	+
		Ct	+	+	++	+++
		A/Cd	++	+++	++	+++
	*yotari*	mm	+	−/+	+	++
		rv/g	++	+	++	+
		Ct	++	+	++	+++
		A/Cd	+	+++	++	+++

+++ strong reactivity; ++ moderate reactivity; + mild reactivity; − no reactivity; mm—metanephric mesenchyme, rv—renal vesicle, g—immature glomeruli, Ct—convoluted tubule, A—ampulla, Cd—collecting duct, E—day of embryonic development; fibroblast growth factor receptor 1 (FGFR1), fibroblast growth factor receptor 2 (FGFR2), receptor-interacting protein kinase 5 (RIP5), and huntingtin-interacting protein 2 (HIP2)

**Table 2:** Staining intensity of specific antibodies in the kidneys of *yotari* and wild-type mice at postnatal days P4, P11, and P14.

Postnatal Day (P)	Animal	Structure	Antibody			
			FGFR1	FGFR2	RIP5	HIP2
P4	wild type	G	+/++	+++	+/++	++
		PCT	−/+	+	+/++	+
		DCT	+	+	+++	++
	*yotari*	G	+	++	+	++
		PCT	+	+	+++	+
		DCT	+	++	+	+
P11	wild type	G	++	+++	++	+
		PCT	++	+	++	++
		DCT	++	+++	++	++
	*yotari*	G	++	++	++	++/+++
		PCT	++	+	++	++
		DCT	++	+/++	++	++
P14	wild type	G	++	+++	+++	++++
		PCT	++/+++	+	++	++
		DCT	++/+++	+++	+++	++
	*yotari*	G	+	+++	+++	++
		PCT	++	+	++	++
		DCT	++	++/+++	+++	++

+++ strong reactivity; ++ moderate reactivity; + mild reactivity; − no reactivity; G—glomeruli, PCT—proximal convoluted tubules, DCT—distal convoluted tubules, P—day of postnatal development.

**Table 3:** Antibodies used for immunofluorescence.

Antibodies		Catalog Number	Host	Dilution	Source
Primary	RIP5 (N-16)	sc-162109	Goat	1:50	Santa Cruz Biotechnology, (Texas, TX, USA)
	Flg (C-15)	sc-121	Rabbit	1:50	Santa Cruz Biotechnology, (Texas, TX, USA)
	Bek (C-17)	sc-122	Rabbit	1:50	Santa Cruz Biotechnology(Texas, TX, USA)
	HIP2 (D27C4) mAb	#8226	Rabbit	1:100	Cell Signaling Technology (CST), (Danvers MA, USA)
	p44/42 MAPK (Erk1/2) (137F5)	CST-4695S	Rabbit	1:250	Cell Signaling Technology (CST), (Danvers MA, USA)
	mTOR	PA5-34663	Rabbit	1:100	Thermo Fisher Scientific (Waltham, MA, USA)
	Human/Mouse/Rat Vimentin Antibody	AF2105	Goat	1:300	R&DSystems (Minneapolis, MN, USA)
	Anti-nephrin Antibody (B-12)	sc-377246	Mouse	1:50	Santa Cruz Biotechnology, (Texas, TX, USA)
Secondary	Anti-Goat IgG, Alexa Fluor^®^ 594	705-295-003	Donkey	1:400	Jackson Immuno Research Laboratories, Inc., (Baltimore, PA, USA)
	Anti-Rabbit IgG, Alexa Fluor^®^ 488	711-545-152	Donkey	1:400	Jackson Immuno Research Laboratories, Inc., (Baltimore, PA, USA)
	Anti-Goat IgG, Alexa Fluor^®^ 488	705-545-003	Donkey	1:400	Jackson Immuno Research Laboratories, Inc., (Baltimore, PA, USA)
	Anti-Mouse IgG, Alexa Fluor^®^ 488	715-545-150	Donkey	1:400	Jackson Immuno Research Laboratories, Inc., (Baltimore, PA, USA)

## Data Availability

All data and materials are available upon request.

## Supplementary Materials

The following supporting information can be downloaded at: https://www.mdpi.com/article/10.3390/ijms23042025/s1.
