# Supplementary material for: Immunohistochemical Expression Pattern of FGFR1, FGFR2, RIP5, and HIP2 in Developing and Postnatal Kidneys of Dab1−/− (yotari) Mice
Source: Int J Mol Sci. 2022 Feb 11;23(4):2025. doi: 10.3390/ijms23042025 (PMC8879463; doi:10.3390/ijms23042025)
Supplement: Supplementary file 1 [file ijms-23-02025-s001.zip › ijms-1546524-supplementary.pdf]

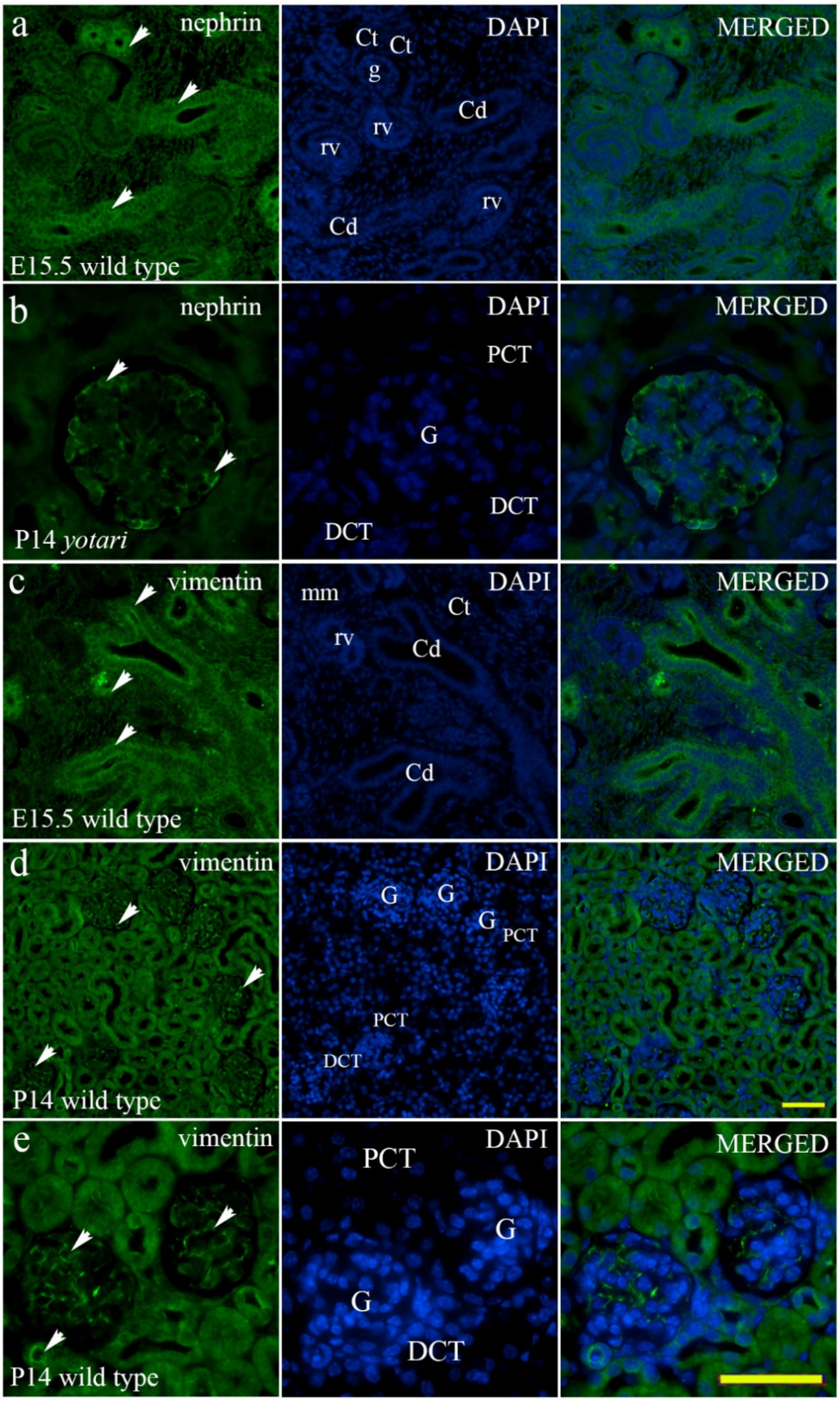

Figure S1. Immunofluorescence staining of embryonic and postnatal wild-type and *yotari* mouse kidneys with the nephrin (**a**,**b**) and vimentin markers (**c**-**e**). Arrows show the expression pattern of nephrin and vimentin in the metanephric mesenchyme (mm), renal vesicles (rv), immature glomeruli (g), convoluted tubules (Ct), ampullae (A), and collecting ducts (Cd), glomeruli (G), proximal convoluted tubules (PCT) and distal convoluted tubules (DCT) indicated on 4′,6-diamidino-2-phenylindole (DAPI) image. Immunoexpression of nephrin, DAPI staining, and merged nephrin and DAPI at embryonic day E15.5 in wild type (**a**) and postnatal day 14 in *yotari* (**b**). Immunoexpression of vimentin, DAPI staining, and merged vimentin and DAPI at embryonic day E15.5 (**c**) and postnatal day

14 (**d, e**) in wild type. Images **a**,**c** and **d** were taken on magnification ×40 and **b**,**e** on ×100. The scale bar is 50 μm, which refers to all images.

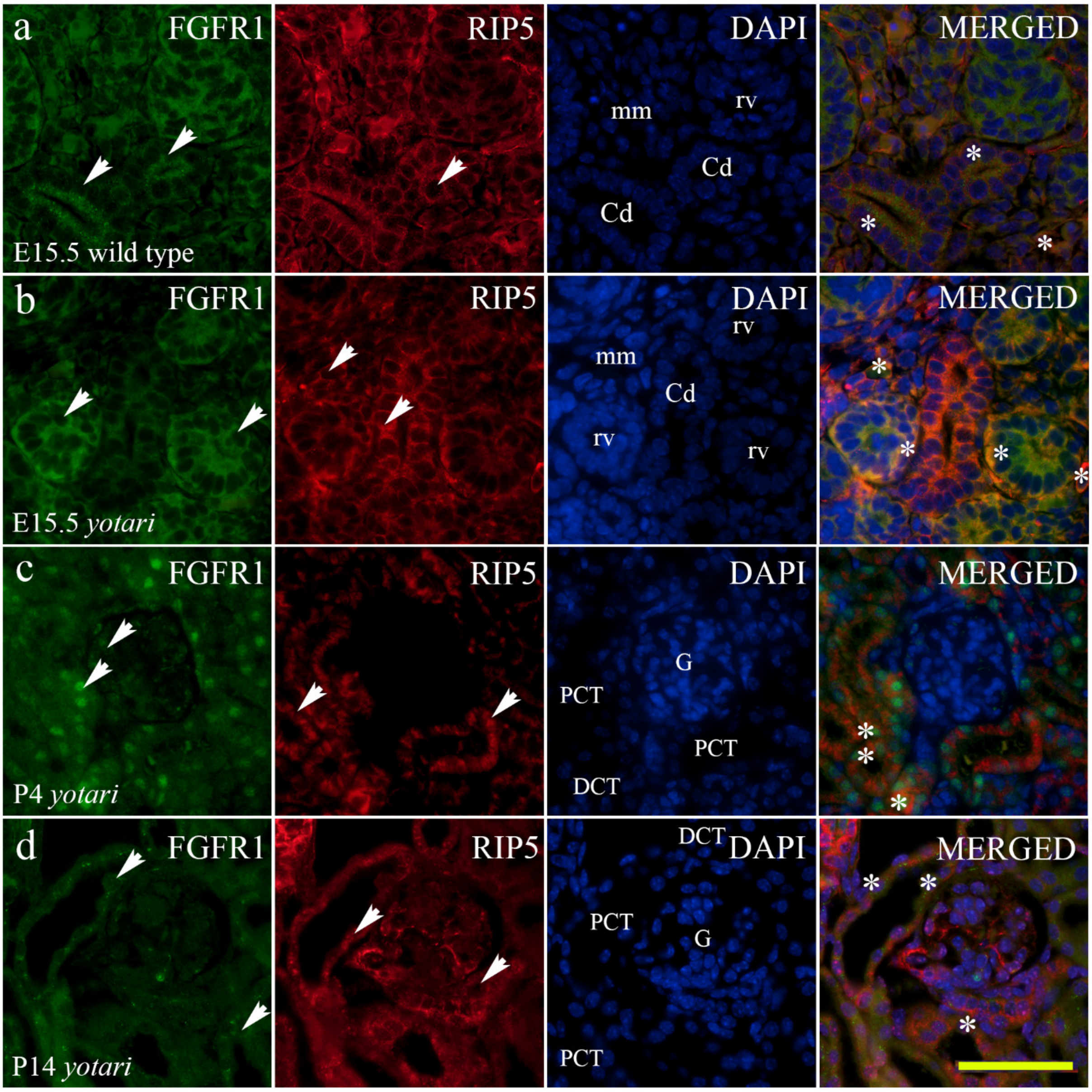

Figure S2. Double immunofluorescence staining of embryonic and postnatal wild-type and *yotari* mouse kidneys with the FGFR1 and RIP5 markers (**a-d**). Arrows show the expression pattern of FGFR1 and RIP5 in the metanephric mesenchyme (mm), renal vesicles (rv), immature glomeruli (g), convoluted tubules (Ct), ampullae (A), and collecting ducts (Cd), glomeruli (G), proximal convoluted tubules (PCT) and distal convoluted tubules (DCT) indicated on 4′,6-diamidino-2-phenylindole (DAPI) image. Immunoexpression of FGFR1, RIP5, DAPI staining and merged FGFR1, RIP5, and DAPI at embryonic day E15.5 in wild type (**a**) and *yotari* (**b**). Immunoexpression of FGFR1, RIP5, DAPI staining and merged FGFR1, RIP5, and DAPI at postnatal day 4 (**c**) and 14 (**d**) in *yotari*. Images were taken on magnification ×100. The scale bar is 50 μm, which refers to all images.

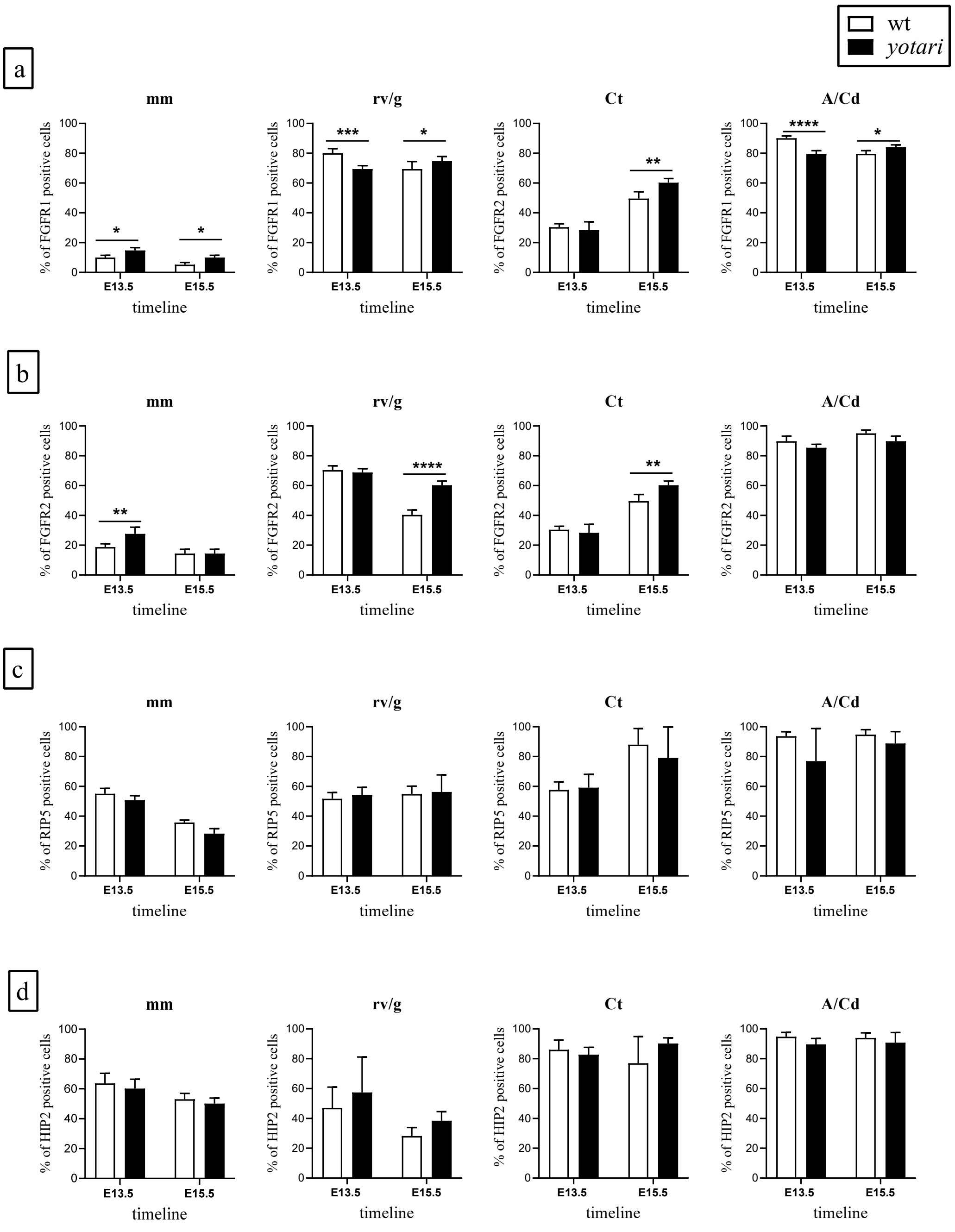

Figure S3. The distribution of the percentages of FGFR1 (**a**), FGFR2 (**b**), RIP5 (**c**), and HIP2 (**d**) positive cells in the metanephric mesenchyme (mm), renal vesicles (rv) or glomeruli (g), convoluted tubules (Ct), and ampulla (A) or collecting ducts (Cd) of wild type and *yotari* kidneys at embryonic days E13.5 and E15.5. Data are presented as the mean ± SD (vertical line) and analyzed by a two-way ANOVA test followed by Tukey's multiple comparison test. Significant differences were indicated by $^{*}p < 0.05$, $^{**}p < 0.001$, $^{***}p < 0.0001$, $^{****}p < 0.00001$. At each time point, ten substructures were assessed.

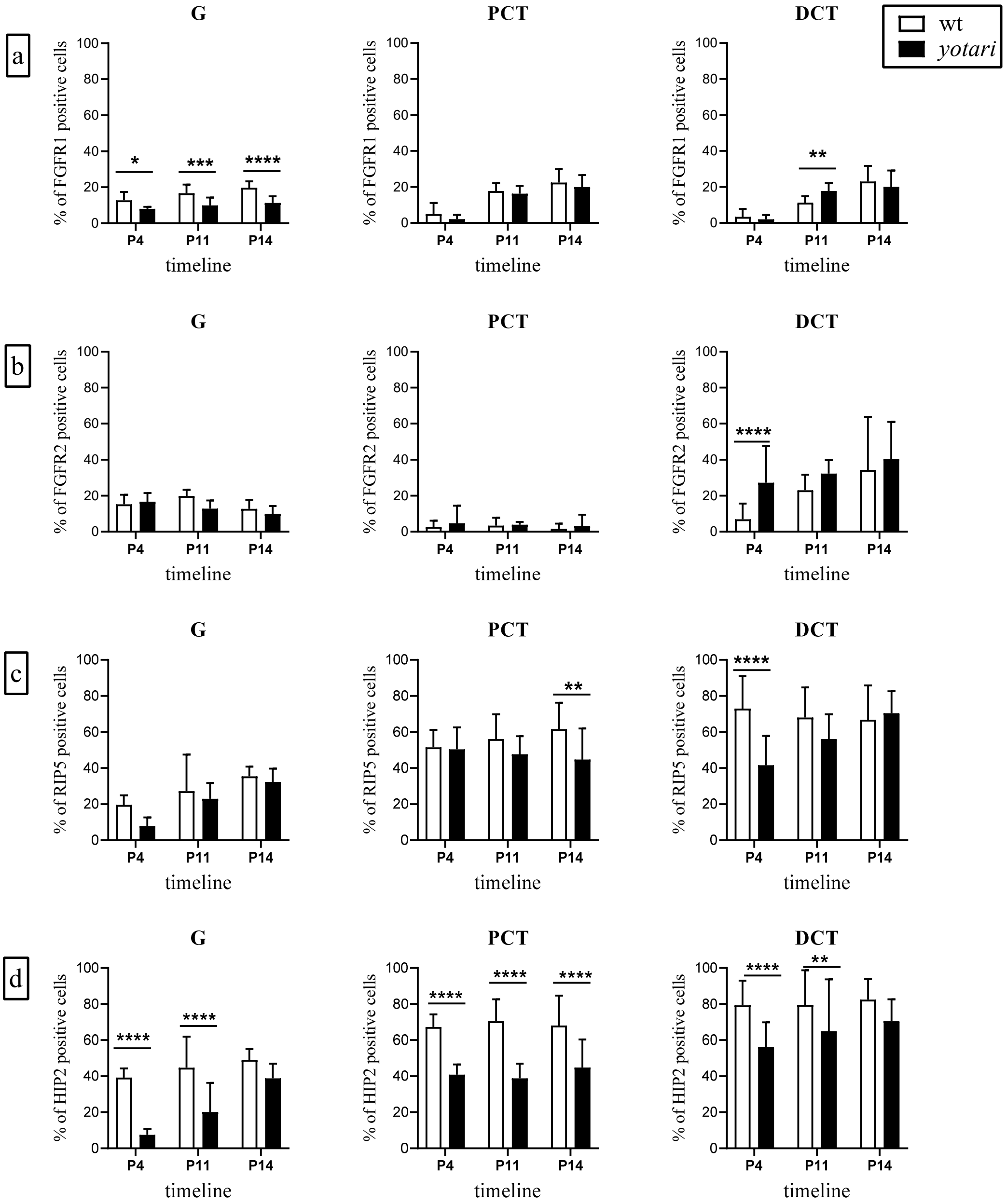

Figure S4. The distribution of the percentages of FGFR1 (**a**), FGFR2 (**b**), RIP5 (**c**), and HIP2 (**d**) positive cells in the glomeruli (G), proximal convoluted tubules (PCT), and distal convoluted tubules (DCT) of postnatal kidneys of wild type and *yotari* animals over time: 4 days (P4), 11 days (P11) and 14 days postnatal (P14). Data are presented as the mean ± SD (vertical line) and analyzed by a two-way ANOVA test followed by Tukey's multiple comparison test. Significant differences were indicated by *$p < 0.05$, **$p < 0.001$, ***$p < 0.0001$, ****$p < 0.00001$. At each time point, twenty substructures were assessed.

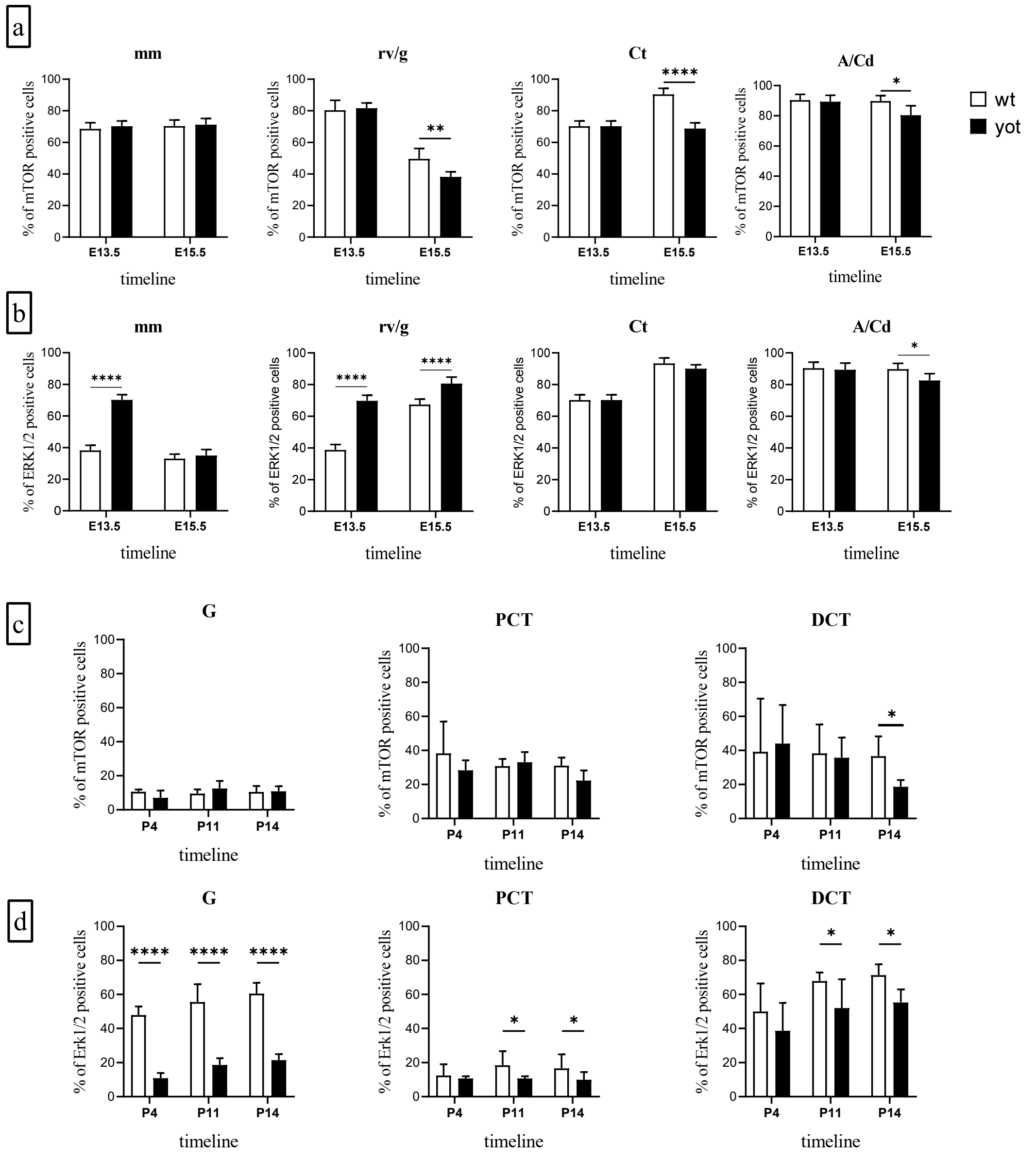

Figure S5. The distribution of the percentages of mTOR (**a**), and Erk1/2 (**b**) positive cells in the metanephric mesenchyme (mm), renal vesicles (rv) or glomeruli (g), convoluted tubules (Ct), and ampulla (A) or collecting ducts (Cd) of wild type and *yotari* kidneys at embryonic days E13.5 and E15.5. The distribution of the percentages of mTOR (**c**) and Erk1/2 (**d**) positive cells in the glomeruli (G), proximal convoluted tubules (PCT), and distal convoluted tubules (DCT) of postnatal kidneys of wild type and *yotari* animals over time (P4, P11, P14). Data are presented as the mean ± SD (vertical line) and analyzed by a two-way ANOVA test followed by Tukey's multiple comparison test. Significant differences were indicated by *$p < 0.05$, **$p < 0.001$, ***$p < 0.0001$, ****$p < 0.00001$. At each embryonic time point, ten substructures were assessed. At each postnatal time point, twenty substructures were assessed.
